# Diagnostic value of HR‐MRI and DCE‐MRI in unilateral middle cerebral artery inflammatory stenosis

**DOI:** 10.1002/brb3.1732

**Published:** 2020-08-07

**Authors:** Guo‐Chang Wang, Yu‐Jing Chen, Xu‐Ran Feng, Ping‐Yong Feng

**Affiliations:** ^1^ Department of Radiology The Second Hospital of Hebei Medical University Shijiazhuang China

**Keywords:** blood–brain barrier, central nervous system vasculitis, DCE‐MRI, HR‐MRI

## Abstract

**Purpose:**

High‐resolution magnetic resonance imaging (HR‐MRI) has high spatial resolution and can simultaneously perform wall and lumen imaging. Dynamic contrast‐enhanced magnetic resonance imaging (DCE‐MRI) can evaluate the integrity of the blood–brain barrier. In this paper, the result of 3.0T HR‐MRI and 3.0T DCE‐MRI has been evaluated to explore the application value of unilateral middle cerebral artery inflammatory stenosis and changes in vascular permeability parameters of stroke events.

**Methods:**

Thirty‐six cases of neurological suspicion of central nervous system vasculitis of our hospital were selected from 20 January 2018 to 1 January 2019, who were diagnosed as unilateral middle cerebral artery M1 stenosis/occlusion by 3D TOF MRA. 3.0T HR‐MRI and 3.0T DCE‐MRI has been applied.

**Results:**

Among the 36 patients who met the inclusion criteria, 23 patients with central nervous system vasculitis were diagnosed. The 23 patients with HR‐MRI showed diffuse thickening and enhanced stenosis. The *K* ^trans^ value of 10/23 patients with acute–subacute cerebral infarction and 3/23 patients in chronic phase were significantly higher than that of the mirror side, and the *K* ^trans^ value of these patients remeasured in the same region of interest is lower than before after 6 months treatment. The *K* ^trans^ value in the target area of 10 patients without cerebrovascular events was not statistically significant compared with the mirror side. The *K* ^trans^ value of patients with acute–subacute cerebral infarction was significantly higher than that without cerebrovascular events (0.098 ± 0.038 vs. 0.007 ± 0.001, *p* = .000), and there was no significant difference between *K* ^trans^ in the chronic infarction group and the other two groups (0.098 ± 0.038 vs. 0.044 ± 0.012, *p* = .058; 0.044 ± 0.012 vs. 0.007 ± 0.001, *p* = .057).

**Conclusion:**

HR‐MRI is an accurate direct imaging method and has a high value for the etiological diagnosis of central nervous system vasculitis. DCE‐MRI could be an effective way to evaluate and monitor blood–brain barrier to prevent clinical ischemic stroke.

## Graphical abstract

In this paper, the result of 3.0T HR‐MRI and 3.0T DCR‐MRI has been evaluated to explore the application value of etiology and changes in vascular permeability parameters of stroke events.

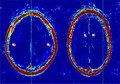

## 1 INTRODUCTION

Intracranial artery stenosis (ICAS) and ischemic stroke (IS) are frequently occurring and primary causes of disability in clinical (Gorelick, Wong, Bae, & Pandey, 2008; Xu et al., 2010). Intracranial atherosclerosis and central nervous system vasculitis are the two most common intracranial stenosis lesions. Moyamoya disease (MMD) is a chronic cerebrovascular occlusive disease with unknown causes, which also found clinical cases usually. Atherosclerosis is a systemic disease which may cause stroke, myocardial, or sudden death (Kramer & Anderson, 2007). Central nervous system (CNS) vasculitis is a vascular inflammation family of rare diseases which can block vessels supplying the brain and spinal cord.

The gold standard for diagnosing vasculitis is currently considered to be dependent on histopathological examination. Due to the peripheral arterioles or the external carotid artery can only be selected, the possibility of vasculitis cannot be ruled out when the histopathological findings are negative (Duna & Calabrese, 1995). Calabrese and Mallek (Calabrese & Mallek, 1988) proposed the diagnostic criteria for primary angiitis of the central nervous system (PACNS), which would include (a) the presence of an unexplained neurologic deficit after thorough clinical and laboratory evaluation; (b) documentation by cerebral angiography and/or tissue examination of an arteritic process within the central nervous system; and (c) no evidence of a systemic vasculitide or any other condition to which the angiographic or pathologic features could be secondary.

MRI of CNS vasculitis showed that the vessel wall was mostly nonfocal stenosis lesions, and the typical lesions showed smooth concentric thickening and obvious enhancement, while the atherosclerotic vessel wall was mostly focal plaques and asymmetrical thickening (Swartz et al., 2009). The clinical symptoms of atherosclerosis are similar with CNS vasculitis, both the manifestations are headache, dizziness, numbness and weakness of limbs, but there are significant differences in the treatment of the vessel wall and lumen structure, treatment options, and prognosis. CNS vasculitis is mainly treated with hormones and/or immunosuppressive agents, while which in intracranial atherosclerosis are drugs to stabilize plaque and activate blood vessels.

Early diagnosis and targeted treatment of CNS vasculitis are particularly important and critical. High‐resolution magnetic resonance (HR‐MRI) technology not only contributes to the diagnosis of central nervous system vasculitis, but also effectively evaluates its therapeutic effect. HR‐MRI can simultaneously perform wall and lumen imaging and accurately assess the degree and extent of vascular stenosis. At the same time, HR‐MRI can provide important information for the determination of the causes of vascular stenosis according to the changes in the structure of vessel wall. This accuracy is very important in differential diagnosis and pathophysiology of cerebrovascular diseases.

The integrity of the collateral circulation is closely related to the occurrence of ischemic stroke, hemorrhage after thrombolysis, early neurological recovery, and long‐term prognosis. The blood–brain barrier (BBB) function can be measured to reflect the establishment of the collateral circulation. Dynamic contrast‐enhanced magnetic resonance imaging (DCE‐MRI) can evaluate the integrity of the BBB by quantitatively calculating vascular permeability parameters which could effectively help the clinical evaluation of the ischemic stroke event, determine the treatment plan, and understand the treatment effect. The core parameter of DCE‐MRI is the transfer constant of the contrast agent from the intravascular to the extracellular space (Volume Transfer Constant, *K* ^trans^), and the unit is min‐1, which can quantitatively reflect the permeability of tiny blood vessels. By tracking the vascular damage dynamic change, DCE‐MRI obtains image information, and processes, and analyzes it through corresponding software to obtain various parameters reflecting the microcirculation of the tissue (Heye, Culling, Valdés Hernández, Thrippleton, & Wardlaw, 2014).

To sum up, the purpose of this study is to apply HR‐MRI to make accurate etiological diagnosis of intracranial arterial stenosis caused by central nervous system vasculitis, so as to guide clinical treatment. At the same time, DCE‐MRI was used to quantitatively analyze the cerebrovascular diseases caused by vasculitis, so as to provide more information for the evaluation of the curative effect and prognosis of patients.

## 2 METHODS

### 2.1 Patients

Thirty‐six cases of neurological suspicion of central nervous system vasculitis were selected from 20 January 2018 to 1 January 2019, at the Department of Medical Imaging, The Second Hospital of Hebei Medical University, who were diagnosed as unilateral middle cerebral artery M1 stenosis/occlusion by 3D TOF MRA. Patients’ Inclusion criteria: First, the results of 3D TOF MRA sequence scan showing unilateral middle cerebral artery (MCA) M1 stenosis/ occlusion; Second, neurological department suspected the cause of disease is because of CNS vasculitis; Third, the patient has a complete clinical data. Patients’ exclusion criteria: First, patients’ internal carotid artery and/ or contralateral middle cerebral artery with obvious stenosis; Second, patients with tumor, hemorrhage, and other central nervous system diseases; Third, patients with poor situation or hard to tolerate MRI; Fourth, patients were allergy to steroid contrast agents; Fifth, image quality is poor which cannot meet the diagnostic requirements; Sixth, patients’ clinical data are incomplete.

### 2.2 Data acquisitions

MRI was performed on a 3.0 Telsa magnetic resonance scanner (Philips Achieva) using an eight‐channel phased array head coil. All patients underwent routine DWI and 3D‐TOF MRA scans. According to the 3D‐TOF MRA MIP image, HR‐MRI T1WI plain sequences were performed parallelly and perpendicularly to the long axis of the stenotic/occlusion vessel; then, DCE‐MRI T1‐Mapping was performed. After the end of the 45‐stage dynamic contract enhanced scanning, the HR‐MRI T1WI enhanced scanning was applied. The specific scan parameters of the above sequence are as follows:

- DWI: TR 2,215 ms, TE 96 ms, matrix of 220 mm × 205 mm, layer thickness of 6.5 mm, layer number of 16, FOV 200 mm × 200 mm, NSA 1, flip angle of 90°;
- MRA: Sequence of 3D‐TOF, TE 3.4 ms, matrix of 396 mm × 255 mm, layer thickness of 0.6 mm, layer number of 152, FOV 200 mm × 200 mm, NSA 1, flip angle of 90°;
- HR‐HRI T1W1 VISTA: TR 800 ms, TE 20 ms, matrix of 332 mm × 301 mm, layer thickness of 0.3 mm, layer number of 70, FOV 200 mm × 181 mm, flip angle of 90°;
- DCE‐MRI: Sequence of Philips e‐thrive, TR 3.2 ms, TE 1.6 ms, matrix of 184 mm × 151 mm, layer thickness of 6.5 mm, layer number of 16, FOV 275 mm × 227 mm, NEX 1; before dynamic enhanced scanning, T1‐mapping was performed with 2°, 5°, 10°, 15°, 20°, 25°, 30°flip angles; the contrast agent of enhanced scan was gadolinium diamine injection(Omniscan, GE Healthcare) 0.1 mmol/kg, artificial rapid injection was carried out through the cubital vein, and then, the vessel was flushed with 15 ml normal saline at a flow rate of 3 ml/s; a total of 45 images were scanned, each with 40 layers.

The scan parameters of enhanced HR‐MRI T1W1 were consistent with HR‐MRI T1WI VISTA plain scan.

### 2.3 Data analyze

Apparent diffusion coefficient (ADC) image and magnetic resonance angiography‐maximum intensity projection (MRA‐MIP) image were obtain from Philips Extended MR WorkSpace 2.6 work station. Measurement and calculation of vascular remodeling were performed on the HR‐MRI T1WI axial image. To find the maximal lumen narrowing (MLN), the image has enlarged to 400%. The cross‐sectional area of the middle cerebral artery at the target and the control was measured and compared, and the normal vascular lumen near the site was used as a control. The blood‐outer membrane boundary in the middle cerebral artery was manually mapped as the vascular area (VA), and the blood‐intimal boundary in the middle cerebral artery was used as the lumen area (LA). The value of the wall area of the middle cerebral artery (Wall Area, WA) is the difference between the area of the blood vessel and the lumen area. Lumen stenosis is the ratio of LA at the target to the LA at the control (Ma et al., 2010). While the ratio is less than 50% as mild stenosis; while the ratio is 50% to 70% as moderate stenosis; while the ratio is larger than 70% as severe stenosis and lager than 90% as occlusion. HR‐MRI can be used to accurately assess the stenosis/occlusion range of MCA: focal stenosis/occlusion refers to lesion range ≤ 1/3 M1 segment, segmental stenosis/occlusion refers to lesion range from 1/3 to 2/3 M1 segment, and full‐length stenosis/occlusion refers to a lesion range ≥2/3 M1 segment.

The image and data processing were using Omini Kinectics (GE Healthcare) software. To complete the conversion of time‐luminance signal to the time‐contrast concentration signal, the original data of 2°, 5°, 10°, 15°, 20°, 25°, and 30° flip angles are, respectively, loaded into the T1‐Mapping calculation. The region of interest (ROI) is manually outlined in the superior sagittal sinus. The ROI is placed at the center of the superior sagittal sinus cross section and must not extend beyond the superior sagittal sinus. The time–concentration curve of the arterial input function (AIF) was obtained by calculation. The extend Tofts linear model was selected as the kinetic model. The model is formalized as follows:

$$C_{t}\left(t\right)=V_{P}C_{P}\left(t\right)+K^{\text{trans}}\int_{0}^{t}C_{P}\left(\tau \right)\exp\left[-\frac{K^{\text{trans}}\left(t-\tau \right)}{V_{e}}\right]d\tau$$

where C_t_(*t*) represents the tissue concentration, V_P_ represents the plasma volume fraction, C_P_(*t*) represents the plasma concentration, V_e_ represents the extravascular extracellular volume fraction, and the core parameter is *K* ^trans^ (volume transfer constant). A region of interest (ROI) with the same size was selected as the research object in the middle cerebral artery blood supply area and the contralateral mirror position of the lesion side. The corresponding parameters were obtained by Omini Kinectics software.

Besides, we grouped the cases according to the different luminal stenosis degree and extent and made a statistical analysis of the relationship between cerebral infarction and the two factors, respectively. The comparisons of the different stages of cerebral infarction and the *K* ^trans^ value were also performed.

### 2.4 Statistical analysis

Statistical analysis of the data was obtained by using the social science (SPSS) version 21.0 for Windows. When measurement data conform to the normal distribution, it was expressed by the mean plus or minus standard deviation and the *t* test was applied. When the measurement data do not satisfy the normal distribution, the median (interquartile range) was applied, and nonparametric two independent samples Wilcoxon's rank sum test and chi‐square test were performed simultaneously. Levene variance analysis and two independent sample *t* test were performed on *K* ^trans^ value. Spearman correlation analysis was used for association between clinicopathological characteristics and factors. The data are expressed in terms of frequency and percentage. Fisher's exact test is used for comparison between the two groups since the total sample size is <40 cases. If the *p* value is <.05, it is considered statistically significant.

## 3 RESULTS

In total, thirty‐six patients were enrolled: twenty‐three patients (16 male patients, mean age 38.7 years; seven female patients, mean age 35.3 years; mean age 37.7 years, age range 16–58 years, median age 40 years) were central nervous system vasculitis. Sixteen of them were positive for cytomegalovirus or herpes simplex virus IgG or both. Two of them were positive for autoimmune antibodies (β‐2 glycoprotein antibody, anticardiolipin antibody, etc.). One of them was diagnosed as Behcet syndrome. The rest are ten atheroscl patients (six male patients, mean age 51.3 years; four female patients, mean age 48.5 years; mean age 50.2 years, age range 33–68 years, median 51 years) and three Moyamoya disease patients (two male patients, mean age 26 years; one female patient, mean age 28 years; mean age 26.7 years, age range 21–31 years, median age 28 years). The infection and immune index are negative for those thirteen patients. The distribution of high‐risk factors for different etiology was described in Table 1.

**Table 1:** Distribution table of high‐risk factors for different etiology

	Central nervous system vasculitis (23 cases)	Atherosclerosis (10 cases)	Moyamoya disease (3 cases)
Age ≥ 55 years	1 (4.3%)	4 (40.0%)	0
Smoking	15 (65.2%)	2 (20.0%)	1 (33.3%)
Drinking	7 (30.4%)	3 (30.0%)	1 (33.3%)
Hypertension	8 (34.8%)	7 (70.0%)	0
Diabetes	3 (13.0%)	4 (40.0%)	0
Hyperlipidemia	4 (17.4%)	5 (50.0%)	0
Heart disease (such as atrial fibrillation)	0	0	0
Family history of ischemic stroke	0	0	0
Infection/immune index	18 (78.3%)	1 (10.0%)	0

**Figure 1:**
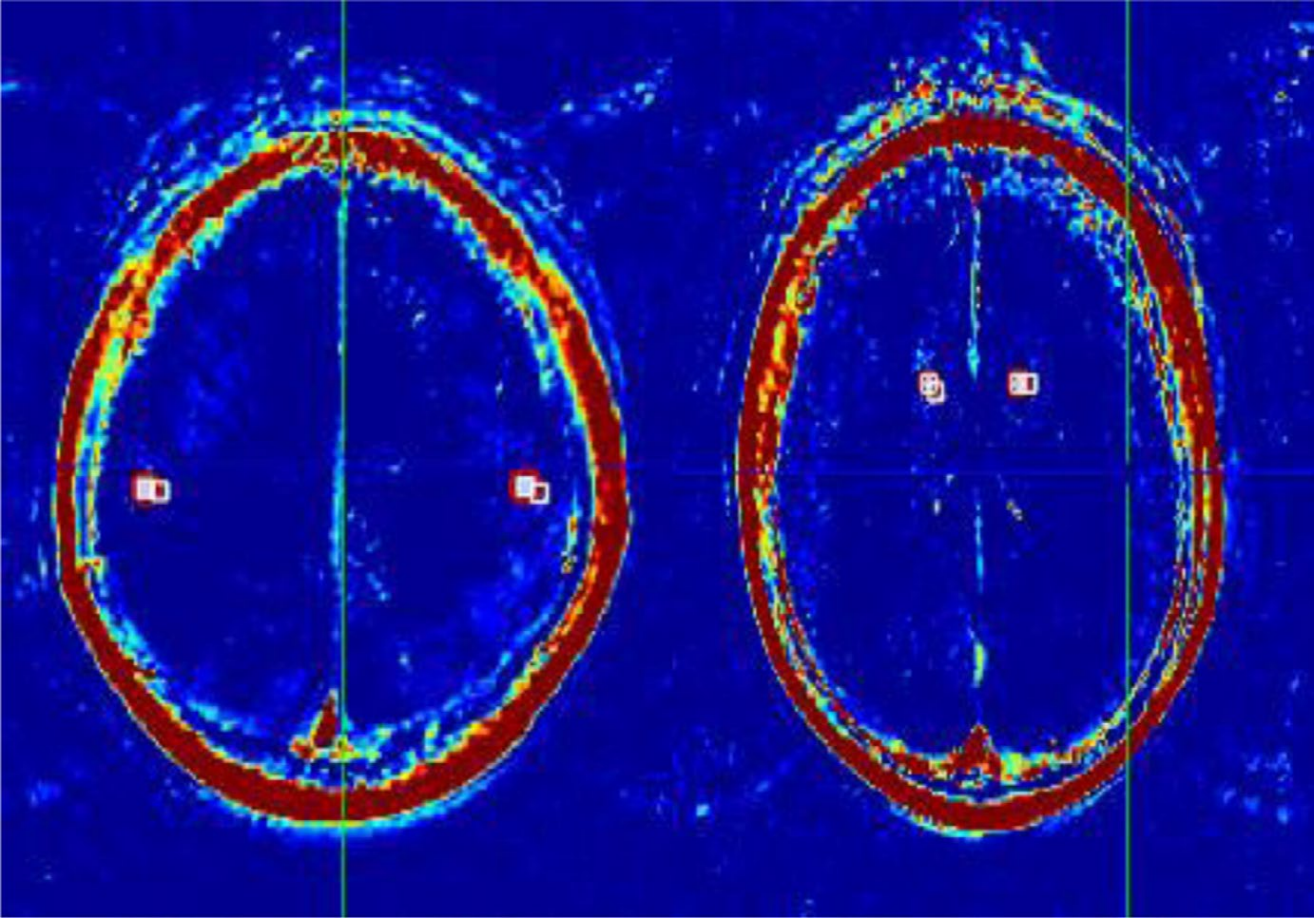
Region of interest (ROI**)** in the MCA blood supply area

The HR‐MRI results of 23 patients with CNS vasculitis showed different degrees of wall thickening and intensification with stenosis of the MCA‐M1 segment (as shown in Figure 1). Of the 23 patients, seventeen (74%) of them were admitted to hospital with different degree of dizziness, speech disorder and limb weakness, etc. Only three (13%) of them developed symptoms such as dizziness, nausea, and vomiting. Among them, five cases (21.7%) had focal lesions (involved range ≤ 1/3 MCA‐M1); 10 cases (43.5%) had segmental lesions (range of 1/3 to 2/3 MCA‐M1); and 8 cases (34.8%) had full‐length lesions (involved range ≥ 2/3 MCA‐M1).

**Figure 2:**
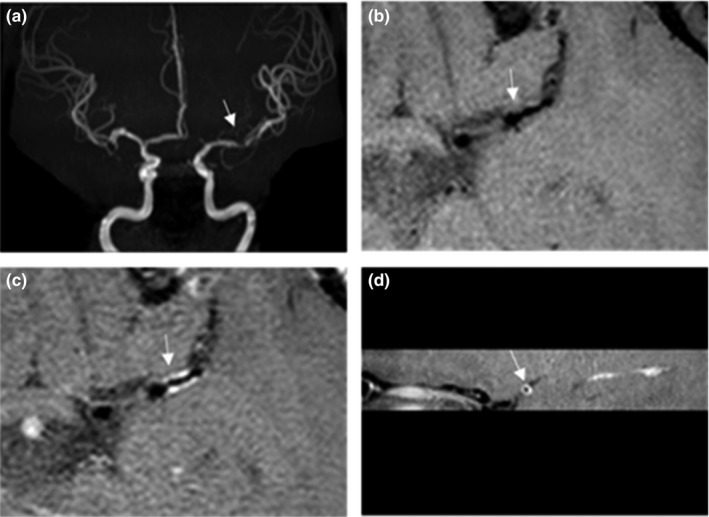
(a) Female, 35 years old, 3D‐TOF MRA shows focal lesions in the M1 segment of the left middle cerebral artery, (b and c) HR‐MRI showed that the wall of the tube was uniformly thickened and strengthened, and (d) the lumen is moderate–severe stenosis

**Figure 3:**
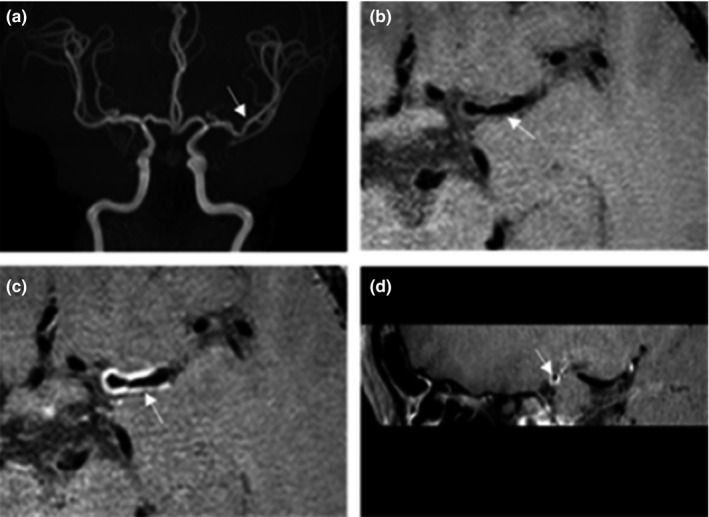
(a) Male, 33 years old, 3D‐TOF MRA showed a slight decrease in the signal of the left middle cerebral artery, (b–d) HR‐MRI shows segmental uniform thickening and strengthening of the wall, and the lumen was estimated to be a mild stenosis

**Figure 4:**
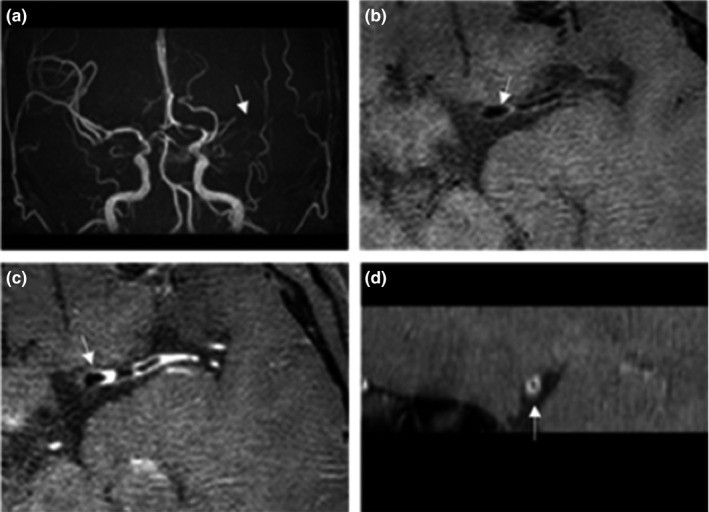
(a) Male 32 years old, 3D‐TOF MRA showed no clear development of the left middle cerebral artery, (b–d) HR‐MRI shows diffuse uniform thickening and strengthening of the wall, and the lumen is moderate–severe stenosis, partial occlusion

**Figure 5:**
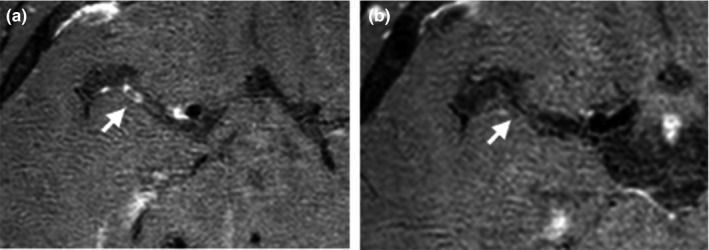
(a) Male, 46 years old, HR‐MRI showed diffuse uniform thickening and strengthening of the M1 segment of the right middle cerebral artery. (b) After treatment, the enhancement of the wall disappeared

Also, eight patients (34.8%) had luminal occlusion (>99%), 13 patients (56.5%) had moderate to severe stenosis (≥50%), and two patients (8.7%) had mild stenosis (<50%). Details of four specific patients’ results were shown in Figures 2, 3, 4, 5. Four patients (17.4%) of the 23 patients had inflammatory lesions involving the internal carotid artery or the contralateral middle cerebral artery or both, causing thickening and strengthening of the wall, but the lumen was not significantly narrow.

In the 23 patients, 10 (43.5%) were in the acute–subacute phase of cerebral infarction, three (13.0%) were in the chronic cerebral infarction, and 10 (43.5%) had no cerebrovascular events. For the 10 patients with acute–subacute cerebral infarction, four (40%) had MCA‐M1 full‐length moderate to severe stenosis/occlusion, four (40%) had MCA‐M1 segmental moderate to severe stenosis/occlusion, and two patients (10%) is focal mild stenosis. For the three patients with chronic cerebral infarction, two (66.7%) had MCA‐M1 segmental moderate to severe stenosis and one (33.3%) had focal moderate to severe stenosis. For the 10 patients with no cerebrovascular events, four (40%) had MCA‐M1 full‐length moderate to severe stenosis/occlusion, four (40%) had moderate to severe stenosis of MCA‐M1, and two (20%) had focal medium to severe stenosis. The distribution is shown in Table 2. The relationship between the difference of degree and extent of vascular stenosis and the occurrence of cerebral infarction was analyzed. The results were not statistically significant (*p* = .253).

**Table 2:** Distribution of stenosis (the degree and range) and cerebrovascular events

	Acute–subacute cerebral infarction	Chronic cerebral infarction	No cerebrovascular events
Focal mild stenosis	2	–	–
Focal moderate to severe stenosis	–	1	2
Segmental moderate to severe stenosis	2	2	4
Segmental occlusion	2	–	–
Full‐length moderate to severe stenosis	1	–	1
Full‐length occlusion	3	–	3

**Figure 6:**
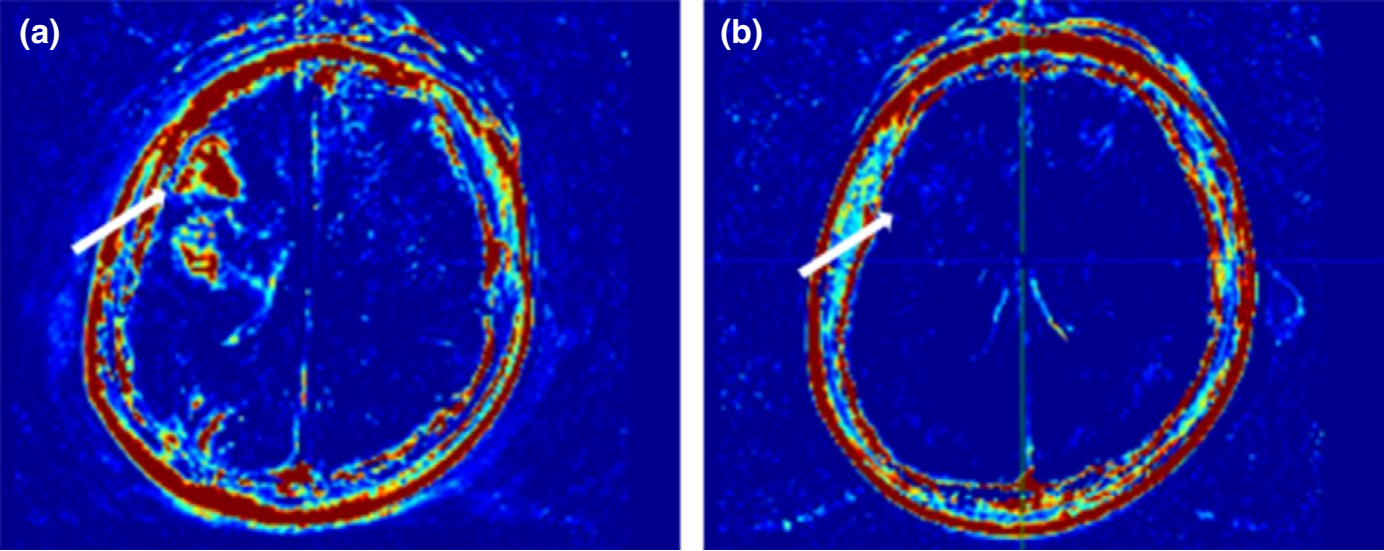
Male, 34 years old, was diagnosed as right middle cerebral artery M1 segmental severe stenosis, right frontal temporal lobe acute cerebral infarction, *K* ^trans^ value is significantly higher. After 6 months of treatment, *K* ^trans^ value reduced

**Figure 7:**
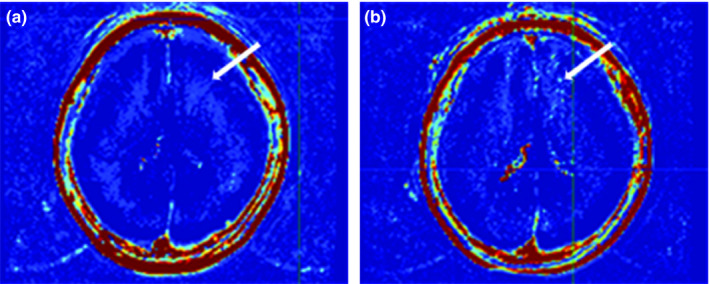
Male, 28 years old, was diagnosed as left middle cerebral artery M1 segmental severe stenosis, no cerebrovascular accident. After 6 months, *K* ^trans^ value raised

For the 10 patients who were in the acute–subacute phase of cerebral infarction, the damage to the blood–brain barrier was significant. Also, the *K* ^trans^ value (0.098 ± 0.038) was significantly higher than the mirror side (0.003 ± 0.001) which considered as statistically significant. And the *K* ^trans^ value (0.039 ± 0.014) was reduced in the same region of interest six months after treatment. For the three patients who were in the phase of chronic cerebral infarction, the *K* ^trans^ value (0.044 ± 0.012) was higher than that of the mirror side. Two of the three patients were reviewed six months after treatment, and the *K* ^trans^ value (0.018 ± 0.009) was lower than before in the same region of interest. For the 10 patients who were without cerebrovascular events, the *K* ^trans^ value (0.007 ± 0.001) was not statistically significant compared with the mirror side. Four of the 10 patients were reviewed six months after treatment, and the *K* ^trans^ value (0.006 ± 0.001) has no significant change compared with the previous one in the same region of interest. Two of the 10 patients had a significantly higher *K* ^trans^ value (0.066 ± 0.019) in the other region of interest than the mirror side. Statistical analysis of the *K* ^trans^ value between the three groups showed that the *K* ^trans^ value of the acute–subacute infarction group was higher than that of the noninfarction group, and there was no statistical difference between the any other two groups (acute–subacute infarction group vs. chronic infarction group vs. noninfarction group: 0.098 ± 0.038 vs. 0.044 ± 0.012 vs. 0.007 ± 0.001, *p* = .000; acute–subacute infarction group vs. chronic infarction group: 0.098 ± 0.038 vs. 0.044 ± 0.012, *p* = .058; acute–subacute infarction group vs. noninfarction group: 0.098 ± 0.038 vs. 0.007 ± 0.001, *p* = .000; chronic infarction group vs. noninfarction group: 0.044 ± 0.012 vs. 0.007 ± 0.001, *p* = .057).The representative images were shown in Figures 6 and 7.

## 4 DISCUSSION

Intracranial artery stenosis (ICAS) and ischemic stroke (IS) are frequently occurring and primary causes of disability in clinical (Gorelick et al., 2008; Xu et al., 2010). CNS vasculitis is an important cause of intracranial artery stenosis and ischemic stroke events. Therefore, early diagnosis and appropriate treatment are important for the prognosis and outcome of this disease. HR‐MRI has a unique display effect on the changes of vessel wall and luminal structure and is of high value for the diagnosis of etiology. HR‐MRI of CNS vasculitis showed diffuse uniform thickening of the vessel wall and showed obvious uniform enhancement on enhanced MRI, and typical manifestation was “orbital signs.” The extent and degree of lumen stenosis had no significant correlation with the occurrence of acute cerebrovascular events. The *K* ^trans^ value in the blood supply area of the distal collateral circulation of the middle cerebral artery stenosis after occlusion was higher than that of the healthy side and was statistically significant, and the *K* ^trans^ value increased more significantly in the case of acute cerebral infarction. After treatment, the *K* ^trans^ value of most patients was reduced, indicating the improvement of microcirculation. However, in a few patients, the *K* ^trans^ value was significantly higher than before, indicating the potential for ischemic stroke.

In this study, 23/36 (63.9%) patients were CNS vasculitis, indicating that CNS vasculitis is an important cause of unilateral middle cerebral artery stenosis/occlusion. Cerebrovascular diseases caused by CNS vasculitis have no obvious specificity on the onset symptoms. The mean age of patients with CNS vasculitis (37.7 ± 10.2) was lower than that of patients with atherosclerosis (50.0 ± 10.4) (*p* = .003). The proportion of smoking in CNS vasculitis was significantly higher than that in atherosclerosis group (*p* = .017), but it was not statistically different from moyamoya disease group (*p* = .286), which may be related to the relevant low number of moyamoya disease group. The distribution of infection or immune indicators (megacytic and/ or herpes virus IgG, autoimmune antibodies, etc.) in the CNS vasculitis distribution rate reached 78.3%, indicating that the incidence of CNS vasculitis is closely related to viral infection and autoimmune response, which consistent with the other literature reports (Hajj‐Ali & Calabrese, 2020; Néel, Pagnoux, Guillevin, & Hamidou, 2012). Therefore, hormones and/or immunosuppressive agents are effective methods for treating CNS vasculitis in our cases.

### 4.1 The results of HR‐MRI

The HR‐MRI finds 23 patients with CNS vasculitis were thickened and strengthened with different degrees of wall obstruction in the MCA‐M1 segment in our study. They were reconstructed vertically and parallel to the direction of the responsible vessel, and the vessel wall was ring‐shaped, with the wall thickening and strengthening more uniform, there is no obvious difference between the front and rear walls and the upper and lower walls; and there are many small branches at the distal end, and some branches can be seen with diffuse mild reinforcement. Li Mingli and other studies of Peking Union Medical College Hospital found that the wall of CNS vasculitis showed diffuse annular thickening with obvious and uniform enhancement, and the wall and lumen structure of the entire affected vessel were narrowed (Li, Xu, Feng, & Jin, 2012); some researchers speculated that this is related to the compression of the vascular lumen by the lining of the lining of the wall after the inflammatory reaction occurs (Gomes, 2010). After the treatment with hormones and/or immunosuppressive agents, the inflammatory reaction is weakened or disappeared, and the wall compression and the stenosis are alleviated or eliminated. This is also confirmed by the inflammatory enhancement of the responsible vessel wall eliminated after treatment with hormones and/or immunosuppressive agents in our study.

It was reported that CNS vasculitis often presents as a single lesion with multiple vessel involvement, and multiple lesions involving a single artery are relatively rare (Hajj‐Ali & Calabrese, 2014). In our cases, most of them were involved in single vessel involvement, which may be related to the chosen of standard unilateral cerebral artery M1 stenosis. In this study, 10 patients were included in the acute/subacute phase of cerebral infarction, three patients with chronic cerebral infarction, and 10 patients without cerebral infarction. The relationship between the extent of stenosis and the degree of stenosis and cerebral infarction was not statistically significant (*p* = .253). There is no clear correlation between the extent and extent of simple stenosis and whether acute ischemic stroke occurs. The occurrence of cerebrovascular accident is the result of multiple factors.

### 4.2 The results of DCE‐MRI

In this study, the vascular permeability parameters of the cerebral arteries in the affected side and the mirror side of the selected subjects were measured. The *K* ^trans^ value (0.098 ± 0.038) of the region of interest in the acute–subacute phase of cerebral infarction was higher than that of the mirror side (0.003 ± 0.001) is significantly elevated; the literature reports that when the acute cerebrovascular accident occurs, the blood–brain barrier is destroyed by a series of complex mechanisms, the contrast agent infiltrates into the intercellular space, and DCE‐MRI captures the microcirculation. The change was characterized by an increase in *K* ^trans^ value; 10 patients were reviewed after 6 months, and the *K* ^trans^ value was reduced in the same region of interest (0.098 ± 0.038 vs. 0.039 ± 0.014, *p* = .016). The *K* ^trans^ value (0.044 ± 0.012) in the three patients (100%) in the chronic phase of cerebral infarction was higher than that in the mirror side; after 6 months, two patients were reviewed and the *K* ^trans^ value was measured in the same region of interest (0.018 ± 0.009) lower than before. The *K* ^trans^ value (0.007 ± 0.001) of the 10 patients (100%) without cerebrovascular events was not statistically significant compared with the mirror side; four patients were reviewed after 6 months, and the *K* ^trans^ value (0.006 ± 0.001) was measured in the same region of interest. No significant change compared with the previous one. This study found the *K* ^trans^ value of the lesion side of the patient gradually decreased over time, suggesting the repair of the blood–brain barrier; however, the *K* ^trans^ value was still higher than the normal level after re‐examination of patients with ischemic stroke. There have reports indicate the self‐repair of the blood–brain barrier is a long process after the cerebrovascular event (Obermeier, Daneman, & Ransohoff, 2013), In this study, it was found that two patients (12.5%) had a higher *K* ^trans^ value (0.066 ± 0.019) in the other region of interest than the mirror side after review. However, the DWI sequence did not find abnormal changes in brain parenchyma in this area; it has been reported in the literature that a significant increase in *K* ^trans^ value suggests the potential for cerebrovascular accidents in this area (Merali, Huang, Mikulis, Silver, & Kassner, 2017). Therefore, follow‐up observation of these two patients is needed. We compared the *K* ^trans^ value of the patients in the acute–subacute infarction group with chronic infarction group and without cerebrovascular events group and found that the acute–subacute infarction would lead to significant destruction of the blood–brain barrier, while the *K* ^trans^ value of the chronic infarction group was not statistically significant compared with the other two groups, which may be related to the fact that there were fewer cases included in the chronic infarction group.

The monitoring of *K* ^trans^ value by DCE‐MRI can microscopically reflect the process of repair and change of blood–brain barrier after acute cerebrovascular events, thus effectively guiding clinical treatment and prognosis, and predicting cerebrovascular accident.

## 5 CONCLUSION

HR‐MRI can provide important diagnostic information for central vasculitis and can accurately assess the degree and extent of vascular stenosis. DCE‐MRI can quantitatively measure the permeability of the blood–brain barrier, so as to reflect the situation of the distal collateral circulation of vascular stenosis, which provides important information for the evaluation of the treatment and prognosis of cerebral infarction, and can predict the possibility of cerebrovascular events to a certain extent.

## CONFLICT OF INTEREST

The authors declare that they have no conflict of interest.

## AUTHOR CONTRIBUTION

Guo‐Chang Wang and Ping‐Yong Feng contributed to the conception and design of the study; Yu‐Jing Chen contributed to the acquisition of data; Xu‐Ran Feng performed the experiments; Ping‐Yong Feng contributed to the analysis of data; Guo‐Chang Wang wrote the manuscript; All authors reviewed and approved the final version of the manuscript.

## ETHICS APPROVAL AND CONSENT TO PARTICIPATE

The study protocol was approved by the Ethics Committee of The Second Hospital of Hebei Medical University. Written informed consent was obtained from all the study subjects before enrollment.

### Peer Review

The peer review history for this article is available at https://publons.com/publon/10.10.1002/brb3.1732.

## Data Availability

The datasets generated and analyzed during the current study are available from the corresponding author on reasonable request.
